# Open surgical implantation of a viable cryopreserved placental membrane after decompression and neurolysis of common peroneal nerve: a case series

**DOI:** 10.1186/s13018-017-0587-y

**Published:** 2017-06-12

**Authors:** E. Rodriguez-Collazo, Y. Tamire

**Affiliations:** 1 Chicago Foot & Ankle Deformity Corrections Center, Department of Surgery Adults & Pediatric Ilizarov Correction, Microsurgical Limb Reconstruction, Presence Saint Joseph Hospital, Chicago, Illinois USA; 2 Medical Science Liaison, Osiris Therapeutics, Inc., 7015 Albert Einstein Drive, Columbia, MD 21046 USA

**Keywords:** Common peroneal nerve injury, Placental membranes, Surgical nerve repair, Foot drop

## Abstract

**Background:**

The purpose of this study is to report on the rehabilitative outcomes associated with common peroneal nerve (CPN) decompression and neurolysis revision when performed with open surgical implantation of a viable cryopreserved placental membrane (vCPM).

**Methods:**

Seven patients who underwent secondary CPN decompression and neurolysis with open surgical implantation of a viable cryopreserved placental membrane (vCPM) after previously failed surgery without vCPM utilization were identified through a retrospective medical record review and outcomes were analyzed. Primary mechanism of injury, severity of symptoms at time of referral, pre-operative and post-operative evaluations on edema with ultrasound, Medical Research Council (MRC) scale for motor strength, range of motion, nerve conduction velocity (NCV), and electromyography (EMG) were analyzed.

**Results:**

Five patients (71.4%) achieved full recovery of motor function MRC grade 5/5, and the remaining two patients achieved MRC grade 4/5. At the 7-month follow-up visit, NCV tests indicated improved conduction velocity and normal amplitude for all 7 patients, and all patients demonstrated proper gait pattern with a return to normal activities of daily living. There were no vCPM-related adverse events.

**Conclusions:**

The use of vCPM wrap as an adjunct to surgical repairs of CPN injuries may contribute to positive clinical outcomes.

## Background

The common peroneal nerve (CPN) is the most frequently injured peripheral nerve in the lower extremity [1–4]. Due to its superficial position on the lateral aspect of the knee and its direct contact with the fibular neck, CPN is vulnerable to injuries secondary to blunt or penetrating trauma, internal or external compression, stretch, contusion, fracture of adjacent bones, lacerations, or other medical conditions [1–4]. CPN neuropathy typically presents with foot drop, motor function deficit, and sensory symptoms, such as pain and paresthesia [3, 5, 6]. Majority of these symptoms can resolve spontaneously or through conservative rehabilitative therapy, however, surgical interventions such as decompression, nerve suture, nerve grafting, and nerve or tendon transfer may be necessary to restore nerve and muscle functions [4, 7, 8].

Although surgical decompression is typically successful, there is a risk of re-entrapment and the development of neuropathy and chronic pain that can contribute to lifelong morbidity. The purpose of this report is to examine, through a case review, the rehabilitative outcomes associated with CPN decompression and neurolysis revision when performed with open surgical implantation of a viable cryopreserved placental membrane (vCPM) (Grafix®PRIME, Osiris Therapeutics, Inc., Columbia, MD).

## Methods

### Population

Following expedited institutional review board approval, Saint Joseph Hospital Protocol #2016-30, data were obtained through a retrospective medical record review of patients who underwent secondary CPN decompression and neurolysis by a single surgeon at the Department of Surgery at Chicago Foot & Ankle Deformity Corrections Center. A total of seven subjects (four males, three females) who had undergone previously failed CPN decompression were identified for analysis. The collection of de-identified data sets included the primary mechanism of injury, severity of symptoms at time of referral, pre-operative and post-operative evaluations on edema with ultrasound, Medical Research Council (MRC) scale for motor strength, range of motion, nerve conduction velocity (NCV), and electromyography (EMG). Descriptive statistics (mean, standard deviation, etc) were used for data analysis.

### Description of viable cryopreserved placental allograft

**Fig. 1:**
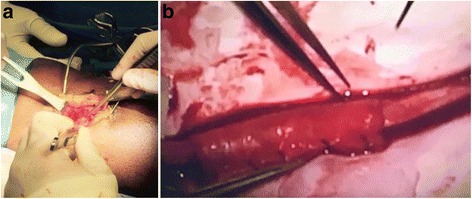
**a** Placement of vCPM around the CPN prior to nerve wrap. **b** Nerve wrap placement after superficial PN internal neurolysis

vCPM is a point-of-care allograft that retains an intact extracellular matrix, resident growth factors, and endogenous neonatal mesenchymal stem cells, fibroblasts, and epithelial cells native to the fresh tissue through a proprietary cryopreservation method [9–11]. vCPM is fully tested according to FDA and AATB requirements and has a 2-year shelf life at −80 °C [12]. vCPMs are ~100-μM-thin membranes that are conforming and self-adherent to other tissues. Human placental membranes have been reported to support natural tissue repair and to reduce inflammation, pain, and scar formation [13, 14]. These properties, make them attractive for various soft tissue reconstructive procedures including microsurgical CPN repairs where minimizing postoperative inflammation, pain, and adhesion formation are critical for successful clinical outcome (Fig. 1a).

### Surgical technique

All procedures were carried out after induction of general anesthesia with surgeon’s utilization of 3.5 loupe magnification for appropriate field visualization. The patients were positioned in lateral decubitus with the affected limb flexed at approximately 35°. After site preparation and draping, a 3-cm lazy “S” shaped transverse incision was made 3 cm distally and 2 cm medially to the fibular head with a 10-blade scalpel. Using Metzenbaum scissors, the incision was carried down to the subcutaneous tissue while protecting vital structures and nerves. Careful blunt dissection by Metzenbaum scissors was performed to separate the muscular fascia and expose the CPN. The CPN was traced around the fibular neck while excising the fascia of the anterior and lateral compartments to decompress the deep peroneal and superficial nerves. A portable nerve stimulator (Checkpoint®; NDI Medical), set at 0.02–2 mA for 2–3 s, was used to locate areas of severe nerve scarring. Microsurgical instruments were used to perform longitudinal and circumferential epineurotomy. Internal and external neurolysis was performed until the bands of Fontana were observed in the fascicles. Over the area of fascicular damage, vCPM allograft was applied (Fig. 1a). Then, the CPN was wrapped with a collagen nerve conduit (Fig. 1b) (NeuraGen®; LifeSciences Corporation). Additional nerve stimulation was performed to ensure no compression was caused by the graft or the conduit. The conduit was transposed laterally and was sutured to the adjacent lateral subcutaneous tissue. The subcutaneous tissue was re-approximated in a layered fashion using a 3-0 Vicryl sutures, and the skin was closed using skin staples. A semicompressive dressing was applied over the incision site, and affected lower extremity was placed in surgical shoe. Patients were placed into immobilization of the affected lower extremity and were provided with post-operative instructions for gradual progression into full weight-bearing ambulation as tolerated.

## Results

Seven patients, three females and four males, were included in this study. The average patient age at the time of surgery was 48 years (range 38–59) (Table 1). Patients presented with foot drop, neurapraxia, and decreased latency. Initial CPN decompressions were attributed to foot and ankle sprain (*n* = 4), total knee arthroplasty (*n* = 2), and trauma to the knee (*n* = 1). Preoperatively, all patients had muscle weakness (MRC grade 1/5). After the revision surgery, 5 (71.4%) of the 7 patients had 100% recovered motor function, MRC grade 5/5, and 2 patients achieved MRC grade of 4/5 (80% recovery) in a mean time of 7 months. Additionally, active range of motion showed a 5-degree increase for both dorsiflexion and plantarflexion in all patients compared to pre-operative assessment. Perineural edema at presentation was resolved by week 12, postoperatively. NCV indicated improved conduction velocity and normal amplitude in each of the cases. All patients resumed proper gait pattern with a return to activity levels of daily living demonstrated prior to injurious event. There were no infections or other adverse events reported related to the use of vCPM. At an average of 16-month follow-up, all patients showed no recurrence of symptoms.

**Table 1:** Mechanism of injury and treatment outcome summary

Patient	Mechanism of injury	Pre-op disposition	Pre-op		Post-op	
			ROM	MRC grade	ROM	MRC grade
1	Sprained foot	- Neurapraxia - Latency - Foot drop	DF: 5 PF:15	1/5	DF: 10 PF:20	5/5
2	Sprained foot	- Neurapraxia - Latency - Foot drop	DF: 8 PF:15	1/5	DF: 13 PF:20	4/5
3	TKA	- Neurapraxia - Latency - Foot drop	DF: 3 PF:10	1/5	DF: 8 PF:15	5/5
4	Trauma to knee	- Neurapraxia - Latency - Foot drop	DF:10 PF:15	1/5	DF:15 PF:20	5/5
5	TKA	- Neurapraxia - Latency - Foot drop	DF: 7 PF:15	1/5	DF: 12 PF:20	4/5
6	Sprained foot and ankle	- Neurapraxia - Latency - Foot drop	DF: 7 PF: 5	1/5	DF: 12 PF: 10	5/5
7	Sprained foot and ankle	- Neurapraxia - Latency - Foot drop	DF: 5 PF:10	1/5	DF: 10 PF:15	5/5
Mean ± SD	N/A	N/A	DF: 6.43 ± 2.30 PF: 12.14 ± 3.93	1	DF: 11.43 ± 2.30 PF: 17.14 ± 3.93	4.71 ± 0.49

*ROM* range of motion, *TKA* total knee arthroplasty, *MRC* Medical Research Council, *DF* dorsiflexion, *PF* plantar flexion, *pre-op* pre-operative, *post-op* post-operative, *SD* standard deviation, *N/A* not applicable

## Discussion

Although majority of CPN injuries can resolve spontaneously, surgical interventions may be necessary to resolve debilitating motor dysfunction, sensory loss, and pain. Surgical manipulation of peripheral nerves, however, is frequently followed by extraneural scar formation and epineural thickening that may lead to chronic compression [15, 16]. The presence of perineural and intraneural scarring and fibrotic adhesions causes physiological obstruction to nerve conduction with subsequent edema and hemorrhage that may interfere with functional recovery of the nerve [15–19]. Furthermore, compared to the outcomes of other peripheral nerves, surgical outcomes of CPN injuries are discouraging [20, 21]. After assessing 28 studies, George et al. reported that functional outcomes of M4 or M5 were obtained in 80% of patients undergoing neurolysis [22]. A 32-year retrospective analysis of 318 patients with knee-level common peroneal nerve lesions by Kim et al. reported that external CPN neurolysis was carried out in 121 (38%) of their patients of which 88% experienced functional outcomes of grade 3 or higher [15]. Seidel et al. also reported that a functionally useful result (M ≥ 4) was produced in 72% of the cases having either external or internal neurolysis and in 28% of the cases with nerve graft [23].

Prevention or reduction of scar and adhesion formation by minimizing postoperative fibrosis is imperative for optimal functional recovery [5, 16, 18, 19]. Various animal models have reported significantly less perineural adhesions and fibrosis in nerves wrapped in human amniotic membrane following neurorrhapy in comparison to control [13, 14]. Based on anti-inflammatory, angiogenic, antimicrobial, and anti-fibrotic properties of placental membranes that may contribute to minimizing postoperative inflammation, pain, and adhesion formation, vCPM allograft was selected for evaluation as an adjunct to augment CPN decompression and neurolysis revision [16–19]. The present study reported the rehabilitative outcomes associated with CPN decompression and neurolysis revision when performed with open surgical implantation of a viable cryopreserved placental membrane. Compared to the outcomes of the previous surgical interventions in these patients, all patients achieved motor function improvement, nerve conduction velocity, and normal amplitude, along with full recovery from foot drop and returned to daily living activities when CPN decompression and neurolysis were in conjunction with the implantation of vCPM. These outcomes were observed in an average of 7 months with no recurrence of symptoms for an average of 16 months compared to unresolved foot drop, neurapraxia, and decreased latency for an average of 16 months after previous unsuccessful CPN decompression and neurolysis.

Limitations of this case series include its retrospective nature, small sample size, and lack of a control group.

## Conclusions

Due to the risk of lifelong morbidity associated with common nerve repair procedures, the efficacy of surgical technique and careful selection and use of an accompanying allograft is paramount. The present study suggests that the use of vCPM wrap as an adjunct to CPN surgery can deliver encouraging results in the recovery of foot drop due to CPN injuries. vCPM may contribute to the natural process of nerve regeneration and repair, with clinical outcomes demonstrated by improved muscle function, nerve conduction velocity, recovery from foot drop, and return to normal activities of daily living in a shorter period.

## Abbreviations

AATB: The American Association of Tissue Banks
CPN: Common peroneal nerve
DF: Dorsiflexion
EMG: Electromyography
FDA: Food and Drug Administration
MRC: Medical Research Council
NCV: Nerve conduction velocity
PF: Plantar flexion
Post-Op: Post-operative
Pre-Op: Pre-operative
ROM: Range of motion
SD: Standard deviation
TKA: Total knee arthroplasty
vCPM: Viable cryopreserved placental membrane
